# CRISPR/Cas9-Induced Double-Strand Break Repair in *Arabidopsis* Nonhomologous End-Joining Mutants

**DOI:** 10.1534/g3.116.035204

**Published:** 2016-11-17

**Authors:** Hexi Shen, Gary D. Strunks, Bart J. P. M. Klemann, Paul J. J. Hooykaas, Sylvia de Pater

**Affiliations:** Department of Molecular and Developmental Genetics, Institute of Biology, Leiden University, 2333 BE, The Netherlands

**Keywords:** *Arabidopsis thaliana*, CRISPR/Cas9, double-strand break, KU80, nonhomologous end-joining

## Abstract

Double-strand breaks (DSBs) are one of the most harmful DNA lesions. Cells utilize two main pathways for DSB repair: homologous recombination (HR) and nonhomologous end-joining (NHEJ). NHEJ can be subdivided into the KU-dependent classical NHEJ (c-NHEJ) and the more error-prone KU-independent backup-NHEJ (b-NHEJ) pathways, involving the poly (ADP-ribose) polymerases (PARPs). However, in the absence of these factors, cells still seem able to adequately maintain genome integrity, suggesting the presence of other b-NHEJ repair factors or pathways independent from KU and PARPs. The outcome of DSB repair by NHEJ pathways can be investigated by using artificial sequence-specific nucleases such as CRISPR/Cas9 to induce DSBs at a target of interest. Here, we used CRISPR/Cas9 for DSB induction at the *Arabidopsis cruciferin 3* (*CRU3*) and *protoporphyrinogen oxidase* (*PPO*) genes. DSB repair outcomes via NHEJ were analyzed using footprint analysis in wild-type plants and plants deficient in key factors of c-NHEJ (*ku80*), b-NHEJ (*parp1 parp2*), or both (*ku80 parp1 parp2*). We found that larger deletions of >20 bp predominated after DSB repair in *ku80* and *ku80 parp1 parp2* mutants, corroborating with a role of KU in preventing DSB end resection. Deletion lengths did not significantly differ between *ku80* and *ku80 parp1 parp2* mutants, suggesting that a KU- and PARP-independent b-NHEJ mechanism becomes active in these mutants. Furthermore, microhomologies and templated insertions were observed at the repair junctions in the wild type and all mutants. Since these characteristics are hallmarks of polymerase θ-mediated DSB repair, we suggest a possible role for this recently discovered polymerase in DSB repair in plants.

Double-strand breaks (DSBs) are one of the most lethal forms of DNA damage. DSBs can occur during normal cellular metabolism or can be induced by external factors, and highly threaten genomic integrity and cell survival (Deriano and Roth 2013). To repair DSBs, cells have two main pathways: homologous recombination (HR) and nonhomologous end-joining (NHEJ). Both of them function together to maintain genome integrity. NHEJ is the predominant pathway in higher eukaryotes and repair may lead to mutations at break sites, such as deletions, insertions, and substitutions. At least two NHEJ pathways have been identified: the classic NHEJ pathway (c-NHEJ) and the backup-NHEJ pathway (b-NHEJ) also called alternative-NHEJ (a-NHEJ) or microhomology-mediated end-joining (MMEJ). The c-NHEJ is initiated by the recognition and binding of the KU heterodimer, consisting of KU70 and KU80 subunits, to DSBs (Walker *et al.* 2001). Once bound to a DSB, the KU heterodimer serves as a scaffold to recruit other c-NHEJ factors to the broken ends and promotes end-joining. Because KU is the key component of the c-NHEJ pathway, this pathway is also called KU-dependent NHEJ. In the absence of KU, other factors gain entry to the DSB site for repair by backup pathways. Although the b-NHEJ pathway was defined by involving multiple components, including poly (ADP-ribose) polymerase 1 (PARP1) the precise mechanism is still not clear (Wang *et al.* 2006). Furthermore, recently PARP1 was shown to be involved in repair of DSBs also in the presence of KU (Luijsterburg *et al.* 2016).

Nowadays, DSBs can be induced artificially at specific sites in the genome by sequence-specific artificial nucleases, which can be used to study DSB repair. These induced DSBs will be mainly repaired via NHEJ, which may lead to targeted mutagenesis. When repair restores the target site for the nuclease, the sequence will be cut again in the continuous presence of the nuclease. This cycle of cutting and repair will continue until incorrect repair destroys the target site. When a homologous sequence such as a sister chromatid, is present, DSB repair may also occur via HR, but this will inevitably also lead to restoration of the target site. A repair template without the target site may be provided by transformation or preinserted in the genome, and, when used for repair, lead to gene targeting (Voytas 2013; Puchta and Fauser 2013a). The current genome editing tool kit comprises four classes of engineered nucleases: modified meganucleases, zinc-finger nucleases (ZFNs), transcription activator-like effector nucleases (TALENs), and the CRISPR/Cas9 (clustered regularly interspaced short palindromic repeat/CRISPR-associated 9) system (Voytas 2013; Puchta and Fauser 2013a; Belhaj *et al.* 2015), of which the CRISPR/Cas9 system is the most easy and straightforward to use.

The CRISPR/Cas9 system is derived from an adaptive immune system present in bacteria and archaea, where it serves in degrading invading foreign plasmid or viral DNA. The type II CRISPR genomic locus encodes the Cas9 (“CRISPR-associated 9”) endonuclease, which can form a complex with two short RNA molecules: CRISPR RNA (crRNA) and *trans*-activating crRNA (tracrRNA), which can be fused into a chimeric single-guide RNA (sgRNA) comprising the functions of both precursor RNAs (Jinek *et al.* 2012). A sgRNA can be assembled to target any DNA sequence, with the prerequisite that a protospacer adjacent motif (PAM) sequence of NGG flanking the 3′ end of the sgRNA target sequence is present, which interacts with the Cas9 PAM interacting domain (PI domain) (Jinek *et al.* 2014; Nishimasu *et al.* 2014).

An *in vitro* study showed that the plant orthologs of PARP1 and PARP2 play a role in backup end-joining, similar to its function in animals (Jia *et al.* 2013). However, the exact role of the PARP proteins in end-joining in plants is still unclear. Previous studies already demonstrated the feasibility of DSB-mediated targeted mutagenesis at artificial and endogenous loci in plants using ZFNs, TALENs, and the CRISPR/Cas9 system (Puchta and Fauser 2013b; Belhaj *et al.* 2015). Here, we investigate the role of KU80, PARP1, and PARP2 in NHEJ by using CRISPR/Cas9 for DSB-mediated targeted mutagenesis at the *Arabidopsis cruciferin 3* (*CRU3*) and *protoporphyrinogen oxidase* (*PPO*) genes. CRISPR/Cas9 nucleases were expressed in wild type and in *ku80*, *parp1 parp2*, and *ku80 parp1 parp2* mutants. Footprint analysis in whole seedlings in the wild type and each of the three mutant genotype backgrounds demonstrated that key factors of NHEJ can affect the outcomes of targeted mutagenesis.

## Materials and Methods

### Plant material

The *ku80* (SALK_016627), *parp1* (GABI-Kat Line 692A05) and *parp2* (SALK_140400) T-DNA insertion lines (ecotype Col-0), the *parp1 parp2* double mutant, and *ku80 parp1 parp2* triple mutant were described previously (Jia *et al.* 2013). More information about these lines can be found at http://signal.salk.edu/cgi-bin/tdnaexpress (Alonso *et al.* 2003).

### CRISPR/Cas9 vector construction and plant transformation

For the CRISPR/Cas9 constructs, oligo’s SP509 and SP510 (*CRU3* target) and SP512 and SP513 (*PPO* target) (Supplemental Material, Table S1) were annealed and cloned in *Bbs*I digested pEn-Chimera (Fauser *et al.* 2014). Subsequently, genes encoding sgRNAs were cloned in pDE-pUbi-Cas9 (Fauser *et al.* 2014) by a Gateway LR reaction, resulting in Cas9-PPO (pSDM3905) and Cas9-CRU (pSDM3903), respectively.

Plant binary vectors were introduced into *Agrobacterium tumefaciens* strain AGL1 by electroporation. *Arabidopsis thaliana* plants of the Col-0 ecotype (wild type, *ku80*, *parp1 parp2*, *ku80 parp1 parp2*) were transformed with T-DNAs containing nuclease expression cassettes, using the floral dip method (Clough and Bent 1998). T1 seeds were grown on MA solid medium without sucrose, supplemented with timentin (100 µg/ml), nystatin (100 µg/ml), and 15 µg/ml phosphinothricin for CRISPR/Cas9 T-DNA selection.

### DNA isolation and footprint analysis

T2 seeds derived from independently selected T1 plants were germinated on 1/2 MS supplemented with 10 µg/ml phosphinothricin for T-DNA selection and pools of 10 seedlings per plant line of 10 d old were disrupted to a powder under liquid N_2_ in a Tissuelyser (Retch, Haan, Germany). Genomic DNA was extracted by the hexadecyl trimethyl ammonuim bromide (CTAB) method (de Pater *et al.* 2009). For predigestion, 1 µg of genomic DNA was digested with *Pst*I (Cas9-CRU analysis) or *Fau*I (for Cas9-PPO analysis) overnight and precipitated. Undigested or predigested DNA was used for PCR with Phusion polymerase (Thermo Scientific) to amplify the nucleases’ target sites, followed by digestion of the PCR products with *Pst*I or *Fau*I and separated in agarose gels. PCR primers are shown in Table S1. Primers SP245 and SP248 were used for the Cas9-CRU target region and primers SP392 and SP538 were used for the Cas9-PPO target region. The resistant fragments were isolated from gel, cloned into pJet1.2 (Thermo Scientific) and sequenced by Macrogen Europe (Amsterdam, The Netherlands). Identical sequences in the same line were considered as one mutagenesis event since they might have resulted from PCR amplification. Two-tailed Mann–Whitney *U*-tests were performed for statistical analysis of deletion and insertion lengths.

### Estimation of relative number of mutations

To estimate the relative number of Cas9-induced mutations the target sites were amplified using undigested genomic DNA. PCR products were digested with the appropriate restriction enzymes and analyzed on agarose gels. The intensity of bands was quantified using ImageJ software. The relative number of mutations was calculated by dividing the intensity of the digest-resistant band by the total intensity of all bands in a given lane (Nekrasov *et al.* 2013).

### High-resolution melting

High-resolution melting (HRM) analyses were performed on PCR clones of undigested DNA from T2 seedlings of wild-type lines Cas9-CRU #2 and Cas9-PPO #7 using Precision Melt Supermix (Bio-Rad), containing EvaGreen saturated dye, and the Bio-Rad C1000 Touch thermal cycler (Bio-Rad). Melt curves were analyzed using the Bio-Rad Precision Melt Analysis software. For the *CRU* target primers SP492 and SP563 were used and for the *PPO* target primers SP560 and SP561 (Table S2). Samples with various melt curves were sequenced by Macrogen Europe (Amsterdam, The Netherlands).

### Data availability

Plasmids and plant lines are available upon request. Figure S1 contains phenotypic data. Figure S2 contains sequences of resistant target sites. Table S1 contains primer sequences. Table S2 contains deletion length distributions. Table S3 contains the number of insertions and templated insertions.

## Results

### DSB-mediated mutagenesis by CRISPR/Cas9 at the CRU3 and PPO loci

**Figure 1:**
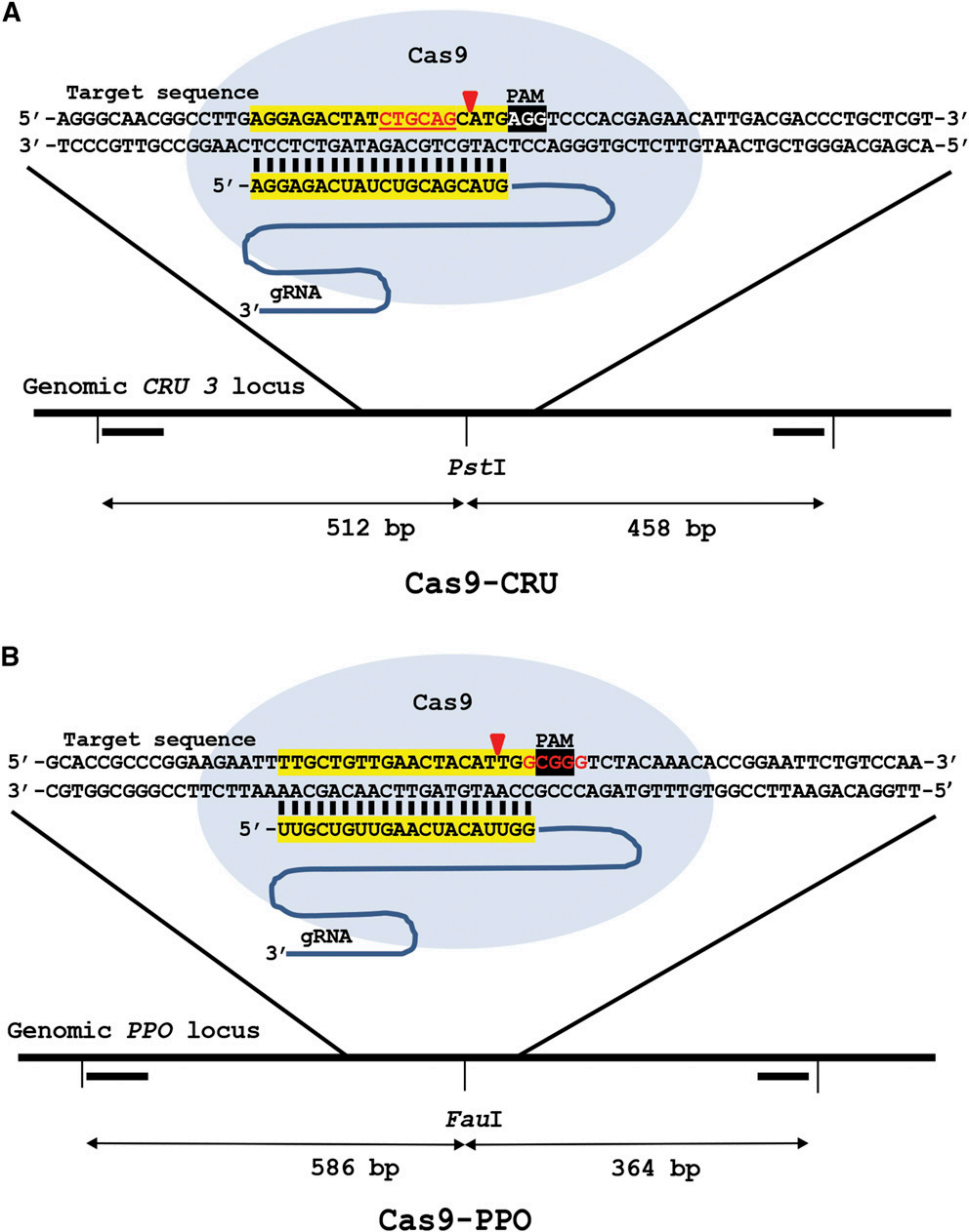
CRISPR/Cas9 endonucleases for DSB induction in *CRU3* and *PPO*. Cas9-CRU (A) with its protospacer in the *CRU3* locus and Cas9-PPO (B) with its protospacer in the *PPO* locus are shown. sgRNA DNA binding sequences are highlighted in yellow, the PAM sequence is highlighted in black and the *Pst*I and *Fau*I restriction sites are shown in red lettering. The primers (▔) used to amplify the target regions and the sizes are indicated. Red arrows indicate the position of DSB induction.

**Figure 2:**
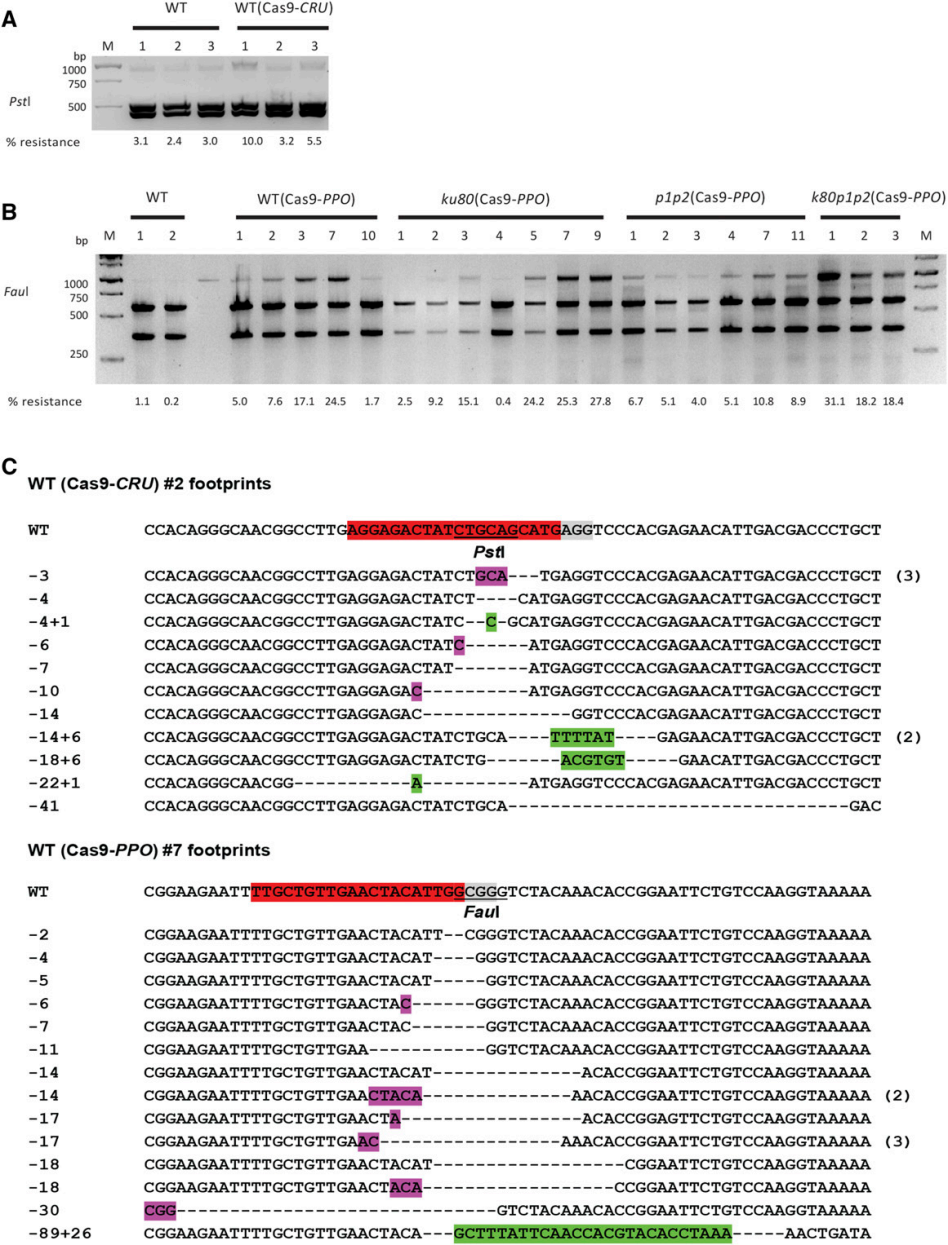
CRISPR/Cas9 endonucleases-induced mutagenesis. (A) The *CRU3* target site was amplified using undigested genomic DNA from untransformed wild-type seedlings and Cas9-CRU-transformed T2 seedlings and digested with *Pst*I. (B) The *PPO* target site was amplified from untransformed wild-type seedlings and Cas9-PPO-transformed T2 seedlings of wild-type and *ku80*, *parp1 parp2* (*p1p2*), and *ku80 parp1 parp2* (*k80p1p2*) mutant plant lines and digested with *Fau*I. *M* is the 1 kb DNA marker, with sizes of the bands at the left, and the % resistant bands is shown below the lanes. (C) Sequences of *CRU3* and *PPO* targets from Cas9-CRU transformant #2 and Cas9-PPO transformant #7. The sgRNA protospacer is in red, PAM sequence is in gray, restriction sites are underlined, deletions are shown by dashes, insertions are in green, and microhomologies used for repair are in purple. Number of multiple clones with the same sequence are shown at the right. Numbers are length of deletions (−) and insertions (+).

In order to investigate repair of induced DSBs, sequence-specific nucleases were designed and expressed in *Arabidopsis*. Wild-type plants were transformed with CRISPR/Cas9 expression constructs via the *Agrobacterium*-mediated floral dip method (Clough and Bent 1998) and T2 transformants were used for further analysis. Nuclease target sites in the *CRU3* and *PPO* genes were selected. The *CRU3* gene encodes a seed storage protein. The *PPO* gene encodes an essential enzyme that is involved in the final step of chlorophyll biosynthesis, and mutagenesis of the *PPO* gene is therefore toxic to plants. Plants expressing nucleases targeted at *CRU3* showed a phenotype similar to wild type, but T2 seedlings of some plant lines expressing Cas9-PPO showed a stunted growth phenotype indicative of homozygous inactivation of the essential *PPO* gene in many cells (Figure S1). To detect mutagenesis caused by nuclease activity and subsequent erroneous NHEJ-mediated DSB repair at the molecular level, T2 seeds of several individual transformants were germinated on phosphinotricin and pooled 10-day-old T2 seedlings were used for DNA isolation and analysis for the presence of NHEJ-induced indels. In order to discriminate DNA molecules with a mutation, PCR products from the region containing the target site were digested with restriction enzymes having a recognition site near the DSB site (*Pst*I for Cas9-CRU and *Fau*I for Cas9-PPO) (Figure 1). Loss of the restriction site as a consequence of erroneous repair resulted in restriction digest-resistant PCR products. After gel electrophoresis, the relative band intensities were measured to estimate the number of mutations in the target sites (Figure 2). Digestion of the PCR products from untransformed wild-type plants left some of the PCR products undigested, probably due to incomplete digestion. However, a distinguishably higher fraction of the PCR products from plant lines transformed with CRISPR/Cas9 nucleases were resistant to enzyme digestion (Figure 2, A and B).

To get a better insight into the mutations induced by the CRISPR/Cas9 nucleases, the restriction digest-resistant PCR products of the *CRU3* and *PPO* targets were cloned and sequenced. Predigested genomic DNA was used for PCR to enrich for mutated sequences. Sequencing revealed mainly deletions and some insertions and substitutions in CRISPR/Cas9 lines (Figure 2C and Figure S2). Short homologous sequences on either the left or right side flanking the deletion were often also present, suggesting MMEJ may have been involved in DSB repair.

**Figure 3:**
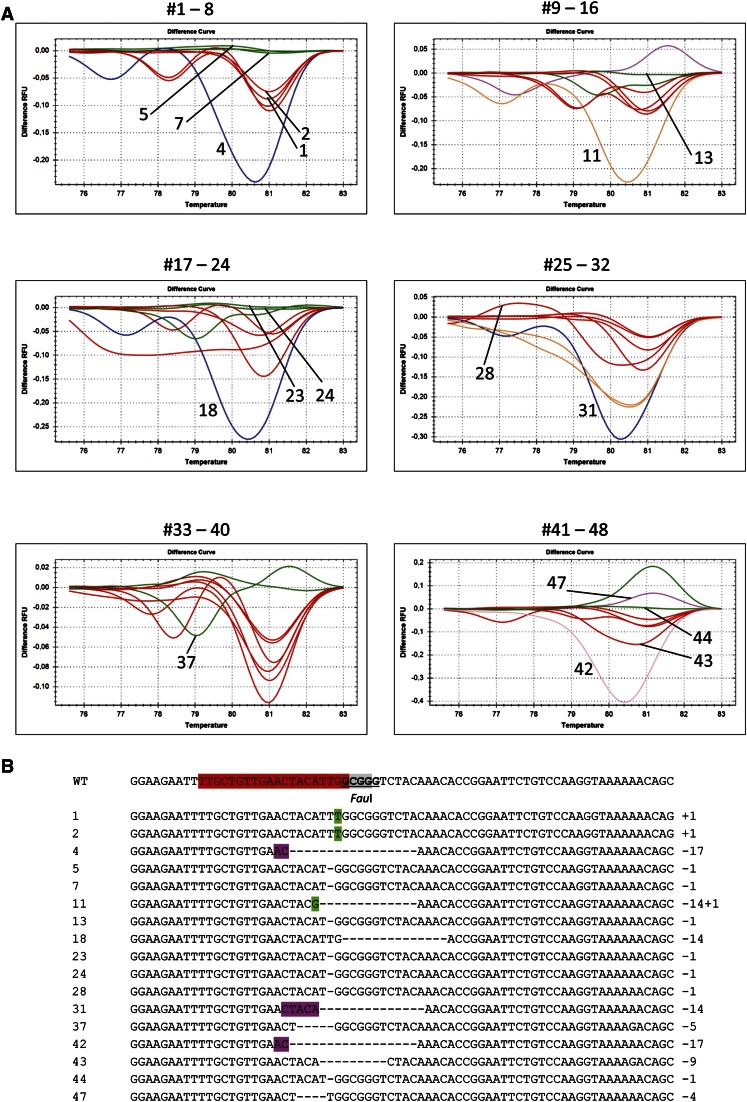
HRM analysis of the *PPO* target. HRM analyses were performed on 48 PCR clones from undigested DNA of a pool of 10 T2 seedlings of wild-type Cas9-PPO transformant #7. (A) Melt curves of samples 1–48 measured in relative fluorescence units (RFU). Numbers indicated in the graph refer to the sequences below. (B) Sequences of representative *PPO* targets. *PPO* sgRNA protospacer (red), the PAM sequence (gray), and *Fau*I restriction site (underlined) are indicated in the wild-type (WT) sequence. Footprints included deletions (dashed lines) and insertions (green). Microhomologies used for repair are shown in purple.

In our experimental design for both Cas9-CRU and Cas9-PPO, the *Pst*I and *Fau*I restriction sites are nearby but do not overlap the DSB site. Therefore, the preselection of loss of a restriction site has the caveat of neglecting mutations that occur outside of the restriction site. To get a more precise insight into the types and frequencies of DSB-mediated mutations in *CRU3* and *PPO*, we used undigested DNA of T2 seedlings for qPCR followed by HRM for footprint analysis. Then indeed, also footprints outside of the restriction site were detected. For Cas9-CRU, HRM was performed on 142 PCR clones from T2 seedlings of line Cas9-CRU #2, which revealed one deletion of 32 bp outside of the *Pst*I site (results not shown). No footprints were detected in the remaining 141 clones, indicating a very low number of mutations in this plant line. For Cas9-PPO, HRM was performed on 48 PCR clones from T2 seedlings of line Cas9-PPO #7, which has a severe phenotype (Figure S1). Nine clones with footprints ranging from 1 bp insertion to 5 bp deletions were found outside the *Fau*I site (Figure 3). None of the 48 clones contained wild-type sequences, indicating a high number of mutations in this plant line.

Taken together, these results show that our CRISPR/Cas9 nuclease constructs are able to induce mutations at the target sites.

### Increased DSB-mediated mutagenesis by CRISPR/Cas9 in c-NHEJ deficient mutants

To compare mutagenesis in wild-type and NHEJ-compromised genetic backgrounds, T-DNA insertion lines *ku80*, *parp1 parp2*, and *ku80 parp1 parp2* as described previously (Jia *et al.* 2012, 2013) were transformed with Cas9-CRU and Cas9-PPO nuclease constructs, and several independent primary transformants were obtained. The target region was PCR amplified using total genomic DNA from pooled T2 10-day-old seedlings of several plant lines as a template, followed by restriction enzyme digestion of the PCR product. The relative band intensity was measured to estimate the amount of mutations for each line. The resistant bands in most of the Cas9-CRU lines were hardly detectable. Clear resistant bands were, however, observed in Cas9-PPO lines (Figure 2B). Plants appeared normal. Apparently, DSB repair of the induced DSBs was still efficient (but less precise) even in the triple mutant *ku80 parp1 parp2*.

### Larger deletions are predominant in c-NHEJ-deficient mutants

To assess the outcomes of DSB repair at the nucleotide level in wild-type and mutant lines, genomic DNA was predigested to enrich for mutated sequences and the resistant bands were purified, cloned, and sequenced. The results showed that there were deletions, insertions, and substitutions at the *CRU3* and *PPO* target sites (Figure S2). The majority of mutations recovered were deletions. Substitutions seem to be very rare events based on the sequenced data and these might be PCR artifacts.

**Figure 4:**
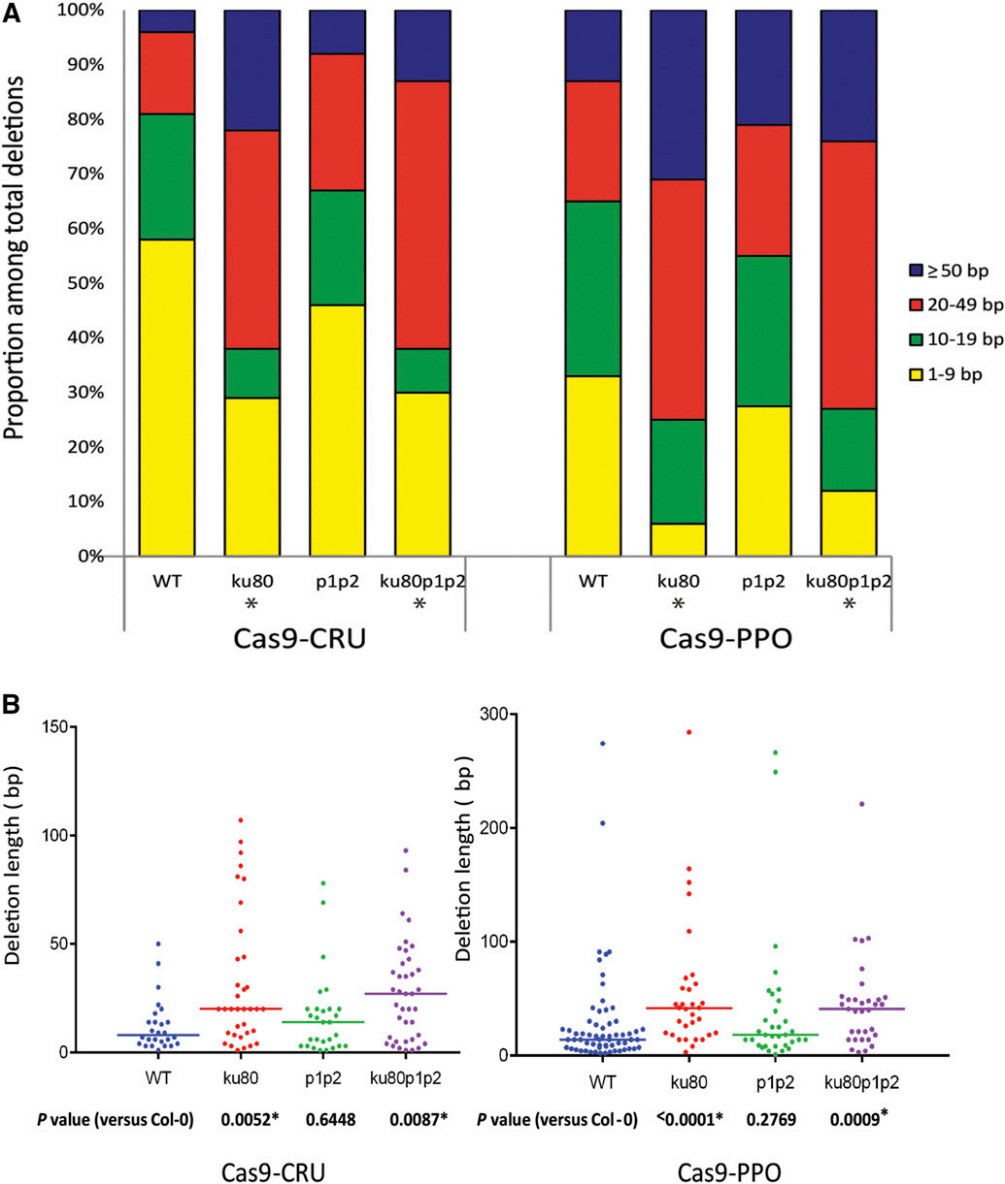
Analysis of deletion length. (A) Distribution of deletion lengths of mutated sequences obtained for the indicated genotypes with Cas9 nucleases. (B) Scatter plot of deletion lengths of the sequences used in (A). Median deletion lengths are indicated by horizontal lines. P-values are derived from a two-tailed Mann–Whitney *U*-test. Asterisks (*) indicate a statistically significant difference from wild type (P < 0.05).

We examined the length of deletions from all genotypes expressing Cas9-CRU or Cas9-PPO (Figure 4A, Figure S2, and Table S3). Due to the loss of restriction site method, 1–2 bp deletions could not be detected in our experimental approach, and only larger deletions that overlap the restriction site were scored. Moreover, each mutation event was scored once for calculating the deletion size average. In wild-type Cas9-CRU transformants, 57% of deletions were <10 bp and about 23% ranged from 10 to 19 bp, 15% ranged from 20 to 49 bp, and 5% were ≥50 bp. The *parp1 parp2* mutant lines showed somewhat longer deletions; about 25% of deletions ranged from 20 to 49 bp and 7% were ≥50 bp. Larger deletions were predominant in the *ku80* and *ku80 parp1 parp2* mutant lines. In *ku80* lines 62% of the deletions were larger than 20 bp (22% were ≥50 bp), and in the *ku80 parp1 parp2* lines 61% of the deletions were larger than 20 bp (12% were ≥50 bp).

Deletion lengths in Cas9-PPO transformants were also examined (Figure 4A). Similar to Cas9-CRU, there were no big differences in deletion length between the wild-type and *parp1 parp2* mutant lines. In the wild type about 33% of deletions were <10 bp, 32% ranged from 10 to 19 bp, 22% ranged from 20 to 49 bp, and 13% were ≥50 bp. In *parp1 parp2* lines 27% of deletions were <10 bp, 27% ranged from 10 to 19 bp, 24% ranged from 20 to 49 bp, and 21% were ≥50 bp. Larger deletions of the *PPO* target were, however, again predominant in *ku80* and *ku80 parp1 parp2* mutant lines. About 75% of deletions in *ku80* lines were larger than 20 bp, and about 73% of deletions in *ku80 parp1 parp2* lines were larger than 20 bp.

We performed statistical analysis using a two-tailed Mann–Whitney *U*-test, to find out whether the observed differences were significant. For the Cas9-CRU and Cas9-PPO nucleases, comparison of deletion lengths in wild type to *ku80* and *ku80 parp1 parp2* lines showed a statistically significant difference, whereas comparison of deletion lengths in wild type to *parp1 parp2* lines did not (Figure 4B). These results indicate that imprecise end-joining after loss of the c-NHEJ key component KU80 resulted in substantial increases in deletion length and suggests a shift to a more error-prone repair mechanism of DSB repair in the absence of KU80.

### Templated insertions in wild-type and NHEJ mutants

**Figure 5:**
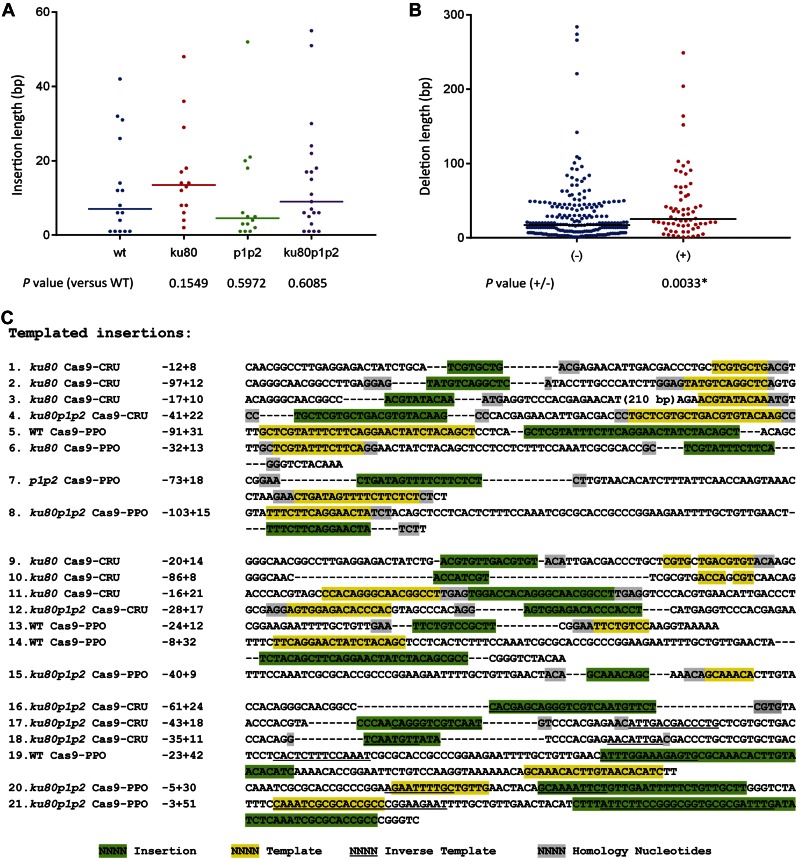
Analysis of insertions. (A) Scatter plot of insertion lengths for the indicated genotypes. Data are combined for both targets. (B) Scatter plot of deletion lengths with (+) or without (−) insertions. Data are combined from all genotypes for both targets. Median insertion or deletion lengths are indicated by horizontal lines. P-values are derived from a two-tailed Mann–Whitney *U*-test. The asterisk (*) indicates a statistical significant difference (P < 0.05). (C) Footprints consisting of deletions (dashes) accompanied by insertions. Insertions are shown in green, template sources for the insertions are shown in yellow (direct strand) or underlined (reverse complement). Homologies between sequences flanking the template and the insertion and probably used as primer are shown in gray. Footprints from 1 to 8 are examples of perfectly matching the template, 9–15 are partially matching the template, and 16–21 are reversely matching the template. Numbers are length of deletions (−) and insertions (+).

Insertion events, sometimes accompanied by deletions, were observed at the target loci in Cas9-CRU and Cas9-PPO transformed wild-type and mutant lines, although less frequently than deletions. The number of insertion events in mutants was comparable to or higher than the number of insertion events in the wild type. More than half of the insertions were smaller than 10 bp. A maximum insertion length of 60 bp was observed. Furthermore, insertion lengths in NHEJ mutant lines were not significantly different from those in wild type when insertion data of both targets were combined, indicating that the insertion mechanism may be independent of KU80 and PARP (Figure 5A). In addition, from the combined data of both targets it can be deduced that the deletion length of junctions with insertions were significantly larger than junctions without insertions (Figure 5B).

Interestingly, many inserted sequences have at least one match to DNA within 100 bp of the repaired DSB. Some insertions have complex compositions with multiple stretches of identity, including reverse complementary homology. These results suggest that polymerase θ may be involved in the repair of these DSBs (Roerink *et al.* 2014). Another signature of Pol θ-mediated DSB repair is the presence of sequence identity between the 3′ end that generated the junction (the primer) and the sequence immediately upstream of the template that is used for DNA synthesis. Such sequence identity is present in about 50% of the inserted sequences (Figure 5C). The *ku80* and *ku80 parp1 parp2* mutant lines appeared to have more templated-insertion events than wild-type and *parp1 parp2* lines, although such insertions were found in all four genotypes (Table S3). Therefore, the templated insertions probably resulted from a KU80- and PARPs-independent alternative end-joining mechanism, such as that mediated by the recently discovered Pol θ (van Kregten *et al.* 2016).

## Discussion

In this study, we demonstrated that CRISPR/Cas9-induced DSBs can be successfully repaired in *Arabidopsis*, even after loss of key components of the NHEJ repair pathway. In general, plants expressing these nucleases look healthy and develop normally (unless an essential gene was targeted). As precise repair restores the break site leading to an intact substrate for the nuclease, a cycle of DSB induction and repair continues until mutations in the target sequence prevent the action of the nuclease. We showed that especially the Cas9-PPO nuclease was a very efficient tool for targeted mutagenesis, with close to 100% mutation of the target site in one line. Since the *PPO* gene is an essential gene, this resulted in stunted growth of the seedlings (Figure S1). This phenotype was not observed in earlier targeted mutagenesis experiments of *PPO* using ZFNs (de Pater *et al.* 2013), indicating a higher activity of CRISPR/Cas9 on the *PPO* gene compared to the ZFNs. We noticed that the sgRNA recognized a sequence in the *PPO* gene with GG just 5′ of the PAM sequence, which was recently shown to promote higher rates of mutagenesis (Farboud and Meyer 2015).

As a result of imperfect end-joining, various mutations in the target sites were found in each line. The DSB sites of our CRISPR/Cas nucleases did not overlap the restriction sites that were used. Therefore, very small deletions or insertions could not be detected in our experimental approach. In the HRM approach we did observe several 1 bp deletions and insertions that were missed by the loss of restriction site method. Such mutations have been found to be present in high frequency in other studies (Fauser *et al.* 2014). However, our method is useful to detect differences between wild type and mutants. In the analysis of DSB repair outcome in NHEJ mutants, we observed a statistically significant increase in the median deletion length at the repair junction in the *ku80* and *ku80 parp1 parp2* mutants compared to wild type, but not in the *parp1 parp2* mutant. This suggests that, in the absence of KU, cells shift to more error-prone end-joining mechanisms. KU is known to competitively bind to DSB ends and protects break ends from end processing (Downs and Jackson 2004; Fell and Schild-Poulter 2015). Thus, when KU is absent, DNA ends are exposed to end resection proteins which would promote the generation of larger deletions. Similar results have been described previously with ZFN-induced DSB repair in a *ku80* mutant and in *ku70* and *lig4* mutants (Osakabe *et al.* 2010; Qi *et al.* 2013).

We previously showed that PARP1 and PARP2 are involved in the MMEJ repair pathway by an *in vitro* end-joining assay in *Arabidopsis* (Jia *et al.* 2013). In the *in vivo* end-joining experiments described here, however, we did not observe a role for PARP1 and PARP2 in MMEJ, and therefore there must be another repair pathway independent of PARP1 and PARP2 that uses microhomology. It is still elusive whether b-NHEJ is a single pathway or a category containing multiple mechanisms (Deriano and Roth 2013). The similar mutation characteristics observed in the *parp1 parp2* mutant and the wild type support the dominant role of the KU-dependent c-NHEJ pathway rather than the PARP-dependent b-NHEJ pathway. Notably, we did not observe much difference in junction characteristics between *ku80* and *ku80 parp1 parp2* mutants, indicating other repair pathways (independent of PARP) with similar characteristics become active when c-NHEJ is absent. However, we cannot rule out that other factors, for example PARP3 (Rulten *et al.* 2011; Langelier *et al.* 2014), could slip into the b-NHEJ pathway without disturbing its outcome.

Insertions were found at both c-NHEJ-proficient and -deficient repair junctions, although most junctions were repaired without an insertion. *ku80* mutants had more insertion events than the wild type. However, the median insertion length in break junctions is a few base pairs and no statistically significant difference was observed among mutants and wild type. Besides, larger deletions (of >20 bp) were found with insertions compared to those in repair products without insertions during Cas9-induced repair. Templated insertions were observed both in c-NHEJ-efficient and c-NHEJ-deficient mutants in animal cells. The current models of templated mutagenesis are based on an MMEJ mechanism involving DNA polymerase θ (McVey and Lee 2008; Koole *et al.* 2014; Roerink *et al.* 2014). In plants, templated insertions were also observed after DSB-induced repair in *Arabidopsis* and tobacco (Shirley *et al.* 1992; Gorbunova and Levy 1997; Salomon and Puchta 1998; Lloyd *et al.* 2012; Vu *et al.* 2014). Furthermore, a recent study showed that the *Arabidopsis* Pol θ ortholog *Tebichi* (*Teb*) is essential for T-DNA integration (van Kregten *et al.* 2016). Templated insertions were found at the repair junctions of T-DNA inserts, and it was shown that *teb* mutants were resistant to T-DNA integration and very sensitive to the DNA-damaging agents bleomycin and MMS. Our results indicate that nuclease-induced DSBs may be repaired by KU-dependent NHEJ, or a backup pathway in the absence of KU, leading to larger deletions in the latter case. Templated insertions, which have the hallmarks of Pol θ-mediated repair, may be formed in both cases, but with a higher frequency in the absence of KU, revealing a complex interplay of repair factors during DSB repair in *Arabidopsis*.

## Supplementary Material

Supplemental material is available online at www.g3journal.org/lookup/suppl/doi:10.1534/g3.116.035204/-/DC1.
