# Supplementary material for: CRISPR/Cas9-Induced Double-Strand Break Repair in Arabidopsis Nonhomologous End-Joining Mutants
Source: G3 (Bethesda). 2016 Nov 17;7(1):193–202. doi: 10.1534/g3.116.035204 (PMC5217109; doi:10.1534/g3.116.035204)
Supplement: Supplementary file 1 [file 193FigureS1.docx]

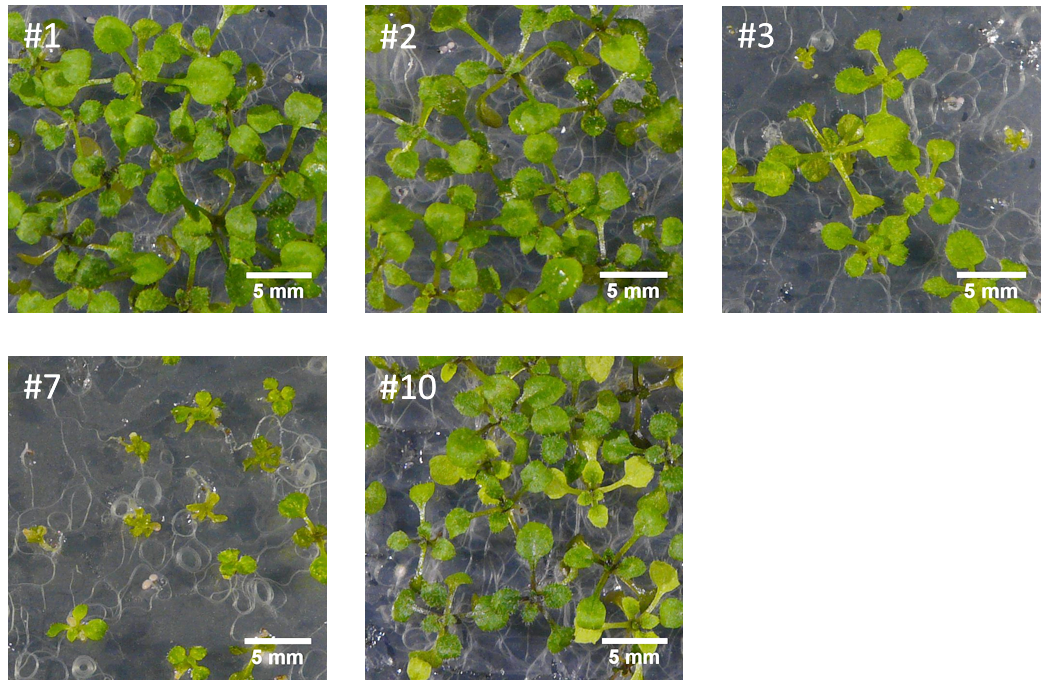


**Figure S1.** Phenotypes of 16 day-old T2 seedlings of 5 independent Cas9-*PPO* transformants. A stunted growth phenotype is observed in some seedlings of lines #3 and #7. The other lines have a phenotype similar to wild-type.
