# Supplementary material for: CRISPR/Cas9-Induced Double-Strand Break Repair in Arabidopsis Nonhomologous End-Joining Mutants
Source: G3 (Bethesda). 2016 Nov 17;7(1):193–202. doi: 10.1534/g3.116.035204 (PMC5217109; doi:10.1534/g3.116.035204)
Supplement: Supplementary file 2 [file 193FigureS2.docx]

**
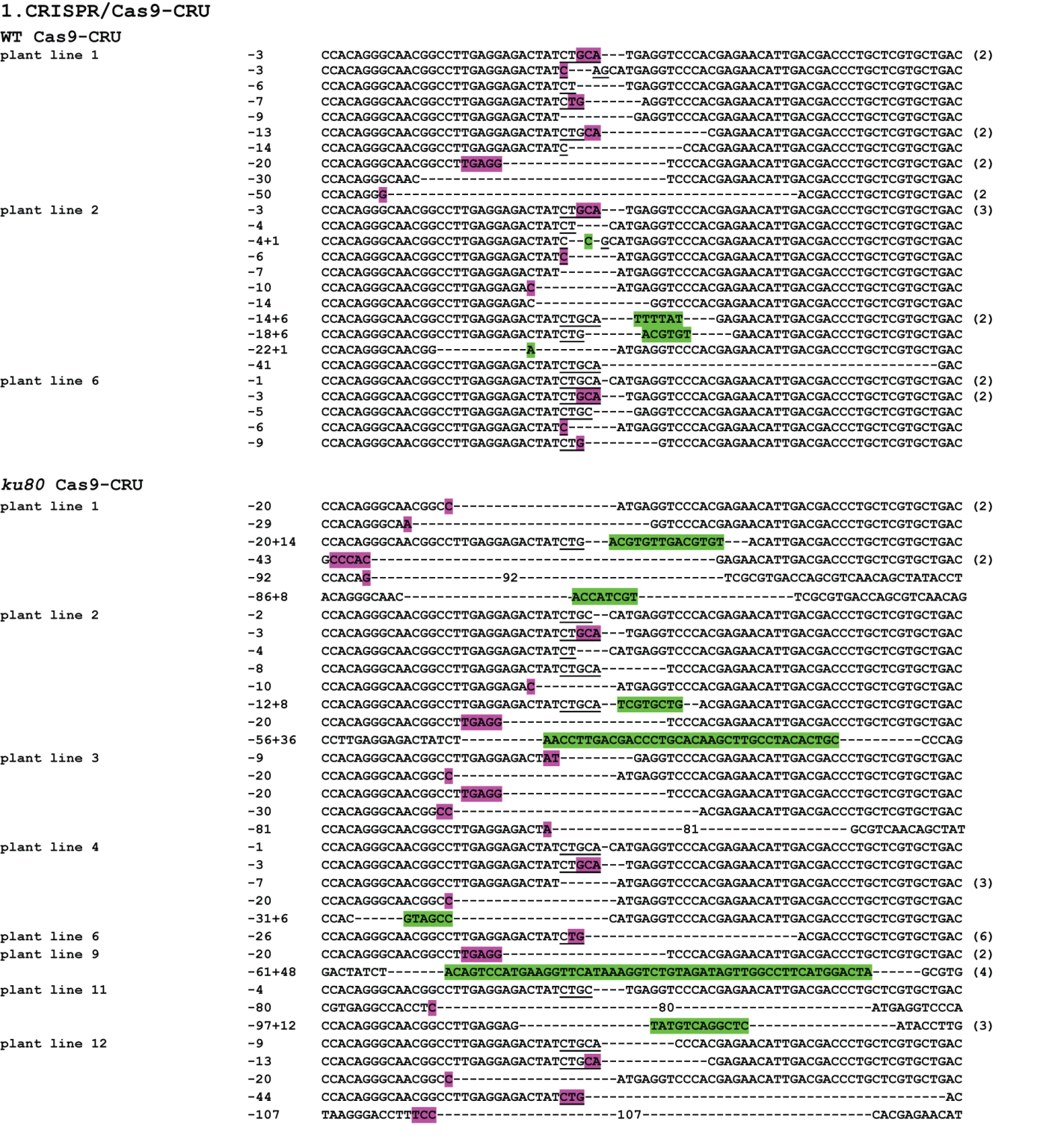
**

**
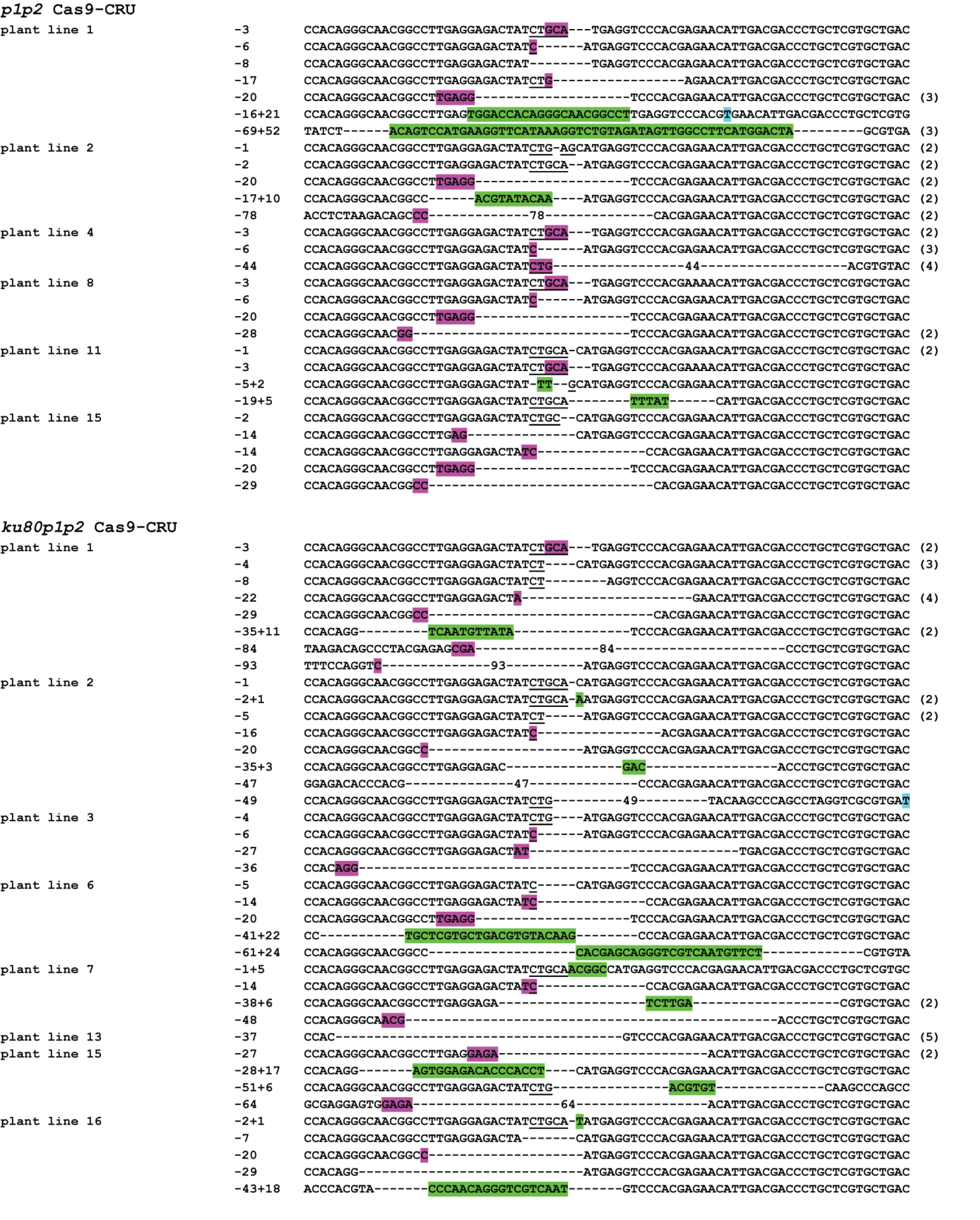
**

**
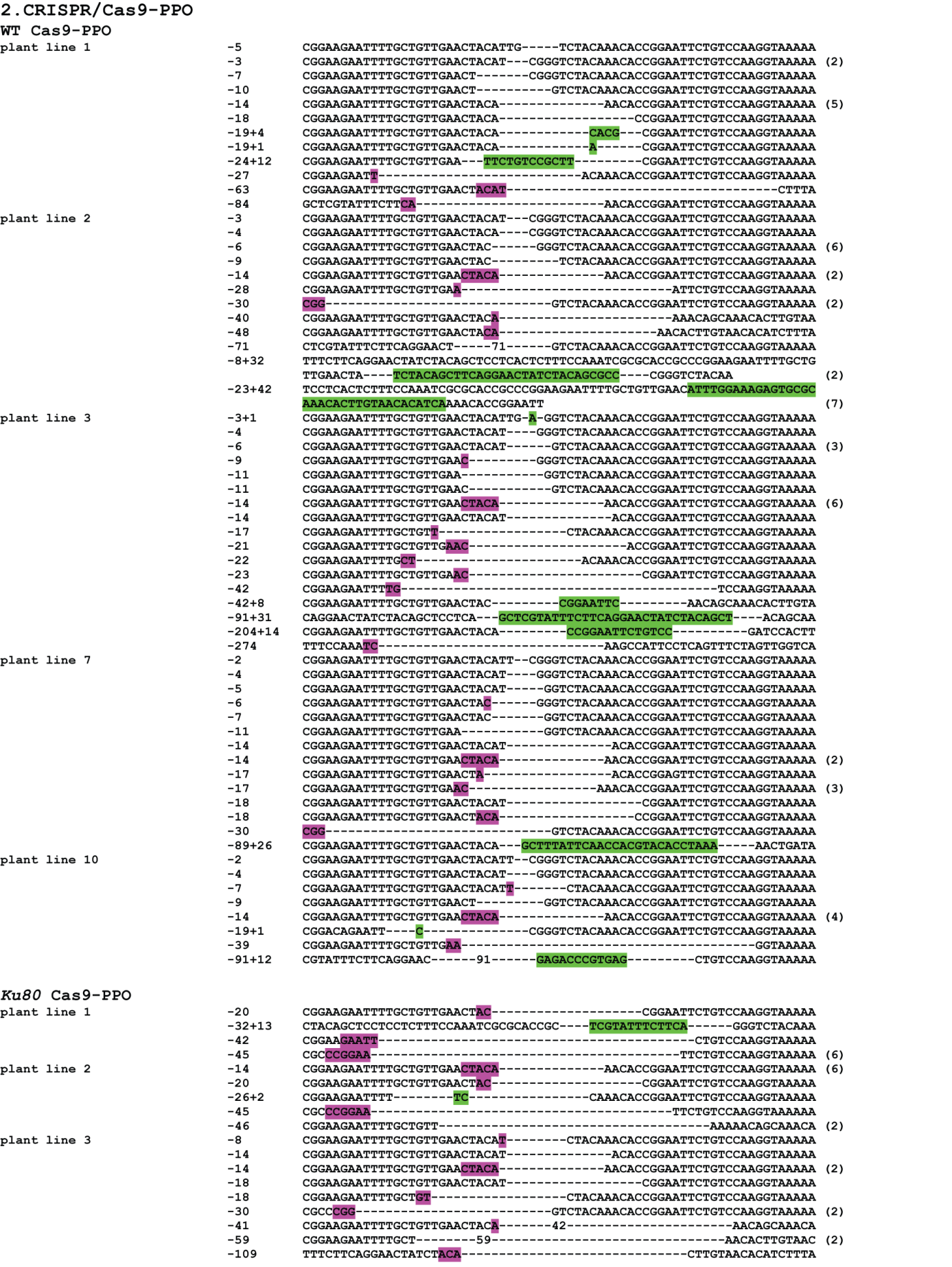
**

**
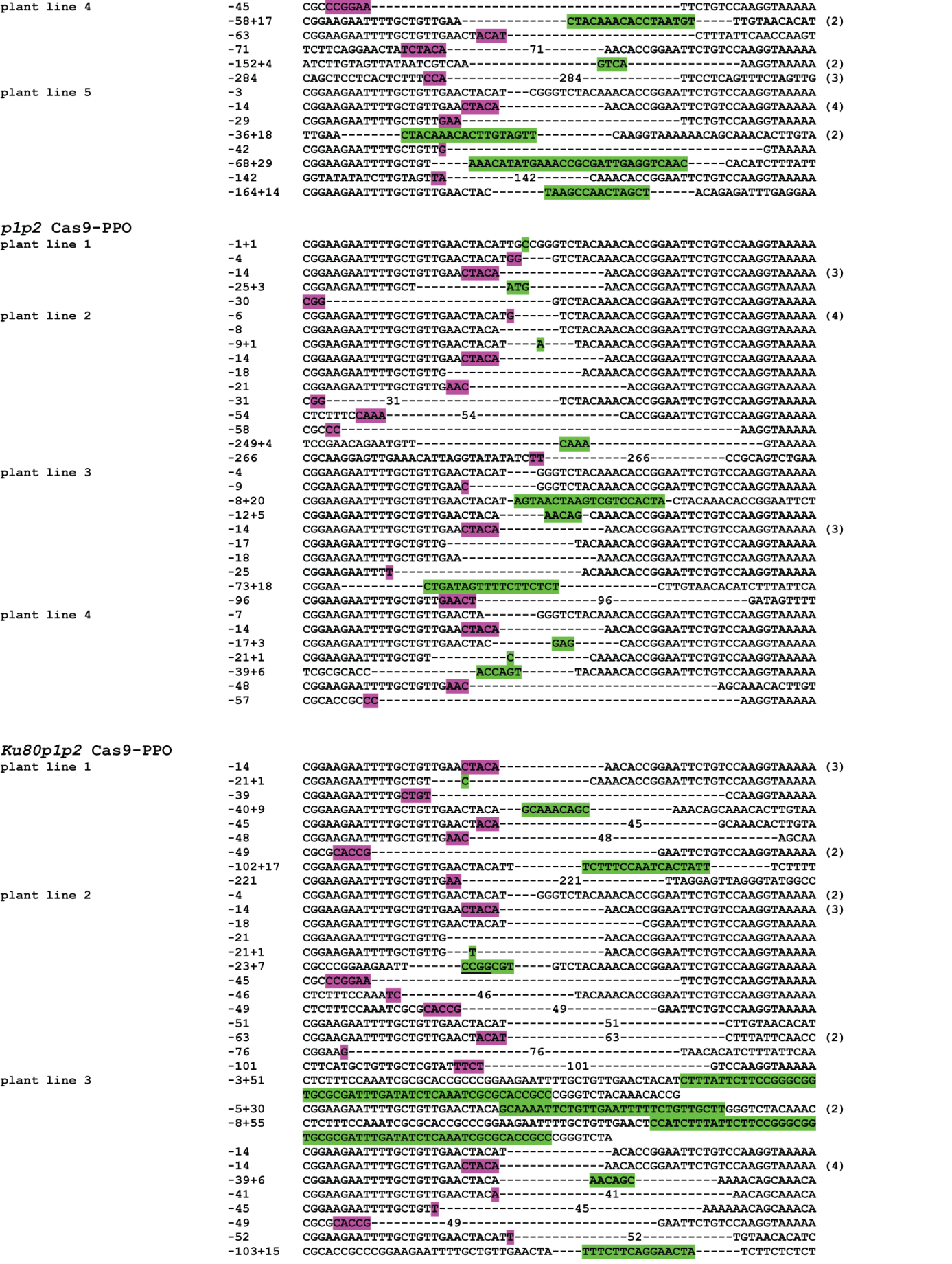
**

**Figure S2**. Sequences of resistant target sites. DNA from several plant lines of wild type and NHEJ mutants with different CRISPR/Cas9 constructs was predigested with the appropriate restriction enzyme (*Pst*I for Cas9-CRU and *Fau*I for Cas9-PPO), used for PCR, digested with the same enzyme and resistant products were cloned and sequenced. Footprints included deletions (dashed lines), insertions (green) and substitutions (blue). Microhomologies used for repair are shown in purple. Number of multiple clones with the same sequence are shown at the right. Numbers are length of deletions (-) and insertions (+).
