# Supplementary material for: CRISPR/Cas9-Induced Double-Strand Break Repair in Arabidopsis Nonhomologous End-Joining Mutants
Source: G3 (Bethesda). 2016 Nov 17;7(1):193–202. doi: 10.1534/g3.116.035204 (PMC5217109; doi:10.1534/g3.116.035204)
Supplement: Supplementary file 3 [file 193TableS1.docx]

**Table S1.** Primers used for cloning and PCR reactions.

| **Primer** | **Sequence** | **Used for** |
| --- | --- | --- |
| SP509 | ATTGAGGAGACTATCTGCAGCATG | sgRNA cloning *CRU3* |
| SP510 | AAACCATGCTGCAGATAGTCTCCT | sgRNA cloning *CRU3* |
| SP512 | ATTGTTGCTGTTGAACTACATTGG | sgRNA cloning *PPO* |
| SP513 | AAACCCAATGTAGTTCAACAGCAA | sgRNA cloning *PPO* |
| SP245 | TGCCAACACTCCAGGCTCTG | *CRU3* target sense |
| SP248 | CAAGTGGTCAACGACAACGG | *CRU3* target antisense |
| SP392 | CACTTTGACAGATTAGGTAG | *PPO* target sense |
| SP538 | CTTCCACTAACTCACCTTC | *PPO* target antisense |
| SP563 | ACCTCTAAGACAGCCCTACG | *CRU3* target sense (HRM) |
| SP492 | TGAGCCTGACATACTCCAAG | *CRU3* target antisense (HRM) |
| SP560 | CTCCTCACTCTTTTCCAAATCG | *PPO* target sense (HRM) |
| SP561 | AGATGTGTTACAAGTGTTTGCTG | *PPO* target antisense (HRM) |
