# Supplementary material for: CRISPR/Cas9-Induced Double-Strand Break Repair in Arabidopsis Nonhomologous End-Joining Mutants
Source: G3 (Bethesda). 2016 Nov 17;7(1):193–202. doi: 10.1534/g3.116.035204 (PMC5217109; doi:10.1534/g3.116.035204)
Supplement: Supplementary file 4 [file 193TableS2.docx]

**Table S2.** Distribution of deletion length for the target site derived from the indicated genotypes.

| Nuclease mutants | Deletion length | | | | Total deletions |
| --- | --- | --- | --- | --- | --- |
|  | 1-9 bp | 10-19 bp | 20-49 bp | ≥50 bp |  |
| WT Cas9-CRU | 15 (57.7%) | 6 (23.1%) | 4 (15.4%) | 1 (3.8%) | 26 |
| *ku80* Cas9-CRU | 10 (28.6%) | 3 (8.6%) | 14 (40%) | 8 (22.8%) | 35 |
| *parp1 parp2* Cas9-CRU | 13 (46.4%) | 6 (21.4%) | 7 (25%) | 2 (7.2%) | 28 |
| *ku80 parp1 parp2* Cas9-CRU | 12 (30.8%) | 3 (7.7%) | 19 (48.7%) | 5 (12.8%) | 39 |
| WT Cas9-PPO | 21 (33.3%) | 20 (31.7%) | 14 (22.2%) | 8 (12.8%) | 63 |
| *ku80* Cas9-PPO | 2 (6.3%) | 6 (18.7%) | 14 (43.7%) | 10 (31.3%) | 32 |
| *parp1 parp2* Cas9-PPO | 9 (27.3%) | 9 (27.3%) | 8 (24.2%) | 7 (21.2%) | 33 |
| *ku80 parp1 parp2* Cas9-PPO | 4 (12.1%) | 5 (15.2%) | 16 (48.5%) | 8 (24.2%) | 33 |
