# Supplementary material for: CRISPR/Cas9-Induced Double-Strand Break Repair in Arabidopsis Nonhomologous End-Joining Mutants
Source: G3 (Bethesda). 2016 Nov 17;7(1):193–202. doi: 10.1534/g3.116.035204 (PMC5217109; doi:10.1534/g3.116.035204)
Supplement: Supplementary file 5 [file 193TableS3.docx]

**Table S3.** Insertions and templated-insertions.

| Nuclease mutants | Total mutations | Insertions | Templated-insertions >3 bp |
| --- | --- | --- | --- |
| WT Cas9-CRU | 26 | 4 (15.4%) | 0 |
| *ku80* Cas9-CRU | 35 | 7 (20.0%) | 6 (17.1%) |
| *parp1 parp2* Cas9-CRU | 28 | 5 (17.9%) | 0 |
| *ku80 parp1 parp2* Cas9-CRU | 39 | 11 (28.2%) | 5 (12.8%) |
| WT Cas9-PPO | 63 | 12 (19.1%) | 4 (6.3%) |
| *ku80* Cas9-PPO | 32 | 7 (21.9%) | 4 (12.5%) |
| *parp1 parp2* Cas9-PPO | 33 | 10 (30.3%) | 1 (3.0%) |
| *ku80 parp1 parp2* Cas9-PPO | 33 | 10 (30.3%) | 6 (18.2%) |
